# Low acclimation capacity of narrow‐ranging thermal specialists exposes susceptibility to global climate change

**DOI:** 10.1002/ece3.4006

**Published:** 2018-04-15

**Authors:** Tricia M. Markle, Kenneth H. Kozak

**Affiliations:** ^1^ Department of Fisheries, Wildlife, & Conservation Biology Bell Museum of Natural History University of Minnesota St Paul MN USA

**Keywords:** acclimation, critical thermal maximum, geographic range, physiological tolerance, salamanders, standard metabolic rate

## Abstract

Thermal acclimation is hypothesized to offer a selective advantage in seasonal habitats and may underlie disparities in geographic range size among closely‐related species with similar ecologies. Understanding this relationship is also critical for identifying species that are more sensitive to warming climates. Here, we study North American plethodontid salamanders to investigate whether acclimation ability is associated with species’ latitudinal extents and the thermal range of the environments they inhabit. We quantified variation in thermal physiology by measuring standard metabolic rate (SMR) at different test and acclimation temperatures for 16 species of salamanders with varying latitudinal extents. A phylogenetically‐controlled Markov chain Monte Carlo generalized linear mixed model (MCMCglmm) was then employed to determine whether there are differences in SMR between wide‐ and narrow‐ranging species at different acclimation temperatures. In addition, we tested for a relationship between the acclimation ability of species and the environmental temperature ranges they inhabit. Further, we investigated if there is a trade‐off between critical thermal maximum (CTMax) and thermal acclimation ability. MCMCglmm results show a significant difference in acclimation ability between wide and narrow‐ranging temperate salamanders. Salamanders with wide latitudinal distributions maintain or slightly increase SMR when subjected to higher test and acclimation temperatures, whereas several narrow‐ranging species show significant metabolic depression. We also found significant, positive relationships between acclimation ability and environmental thermal range, and between acclimation ability and CTMax. Wide‐ranging salamander species exhibit a greater capacity for thermal acclimation than narrow‐ranging species, suggesting that selection for acclimation ability may have been a key factor enabling geographic expansion into areas with greater thermal variability. Further, given that narrow‐ranging salamanders are found to have both poor acclimation ability and lower tolerance to warm temperatures, they are likely to be more susceptible to environmental warming associated with anthropogenic climate change.

## 1 INTRODUCTION

Species with extensive latitudinal distributions generally experience a greater range of thermal conditions than narrow‐ranging species and are anticipated to possess broader physiological tolerances (Bozinovic, Calosi, & Spicer, 2011; Janzen, 1967; Stevens, 1989; Sunday, Bates, & Dulvy, 2011). Broader tolerances may include physiological plasticity (such as thermal acclimation), which enables an animal to express a wider range of thermal sensitivities (Brattstrom, 1968; Feder, 1978; Spicer & Gaston, 1999). Thermal acclimation provides a reversible physiological adjustment in response to environmental conditions and offers an energetic advantage over individuals that experience a time lag in physiological response (Angilletta, 2009). Species with restricted geographic distributions and a narrower range of thermal extremes (e.g., mountaintop endemics) may have less opportunity to evolve thermal acclimation and could be considered thermal specialists for their particular temperature range (Bernardo & Spotila, 2006; Brattstrom, 1968; Gifford & Kozak, 2012; Huey & Kingsolver, 1993). As mean absolute range in temperature is found to increase with increasing or decreasing latitude from the equator (Müller, 1982), a relationship between thermal acclimation ability and latitudinal extent would provide support for the climate variability hypothesis (i.e., a positive relationship should exist between physiological‐tolerance breadth and thermal range of the environment) (Stevens, 1989). Further, if acclimation ability is more prevalent among wide‐ranging species, it could underlie the large disparity in geographic range size found among closely related, ecologically similar species (Cadena et al., 2012; Calosi, David, Bilton, & Spicer, 2008; Feder, 1978; Prosser, 1975).

Temperature can have a strong influence on physiology and metabolic rate (Bennett & Dawson, 1976; Hochachka & Somero, 1973), and can affect numerous life‐history traits including maintenance, growth rate, reproduction, and development (Angilletta, 2009; Angilletta, Niewiarowski, & Navas, 2002; Huey & Stevenson, 1979). To test for an acclimation response to temperature in ectotherms, differences in oxygen consumption (standard metabolic rate, SMR) can be compared for individuals acclimated at lower and higher temperatures (Feder, 1978; Fitzpatrick, 1973; Fitzpatrick & Atebara, 1974). As oxygen uptake is a proxy for ATP demand, changes in SMR can indicate changing energy needs and the health of an individual. After short‐term exposure to a warmer temperature, species with acclimation ability should maintain or slightly increase SMR, indicating a positive response shift in physiology (Feder, 1978; Hillman, Withers, Drewes, & Hillyard, 2009). The ability for individuals to reduce or negate the energetic stresses on metabolism, and to remain active at higher temperatures, could lead to the acquisition of greater resources, increased mating opportunities, and expanded distributions (Bernardo, Ossola, Spotila, & Crandall, 2007; Feder, 1976; Hillman et al., 2009). A drop in metabolic rate (i.e., metabolic depression) at higher temperatures could indicate physiological stress, and without acclimation, the survivorship and fitness of individuals could be profoundly reduced during rapid or prolonged changes in temperature (Bernardo & Spotila, 2006).

A better understanding of how temperature influences the physiology of organisms, and the relationship between physiological variation and large‐scale biogeographic patterns, is also critical in predicting large‐scale responses of species to climatic shifts (Addo‐Bediako, Chown, & Gaston, 2000; Buckley & Huey, 2016; Ghalambor, Huey, Martin, Tewksbury, & Wang, 2006; Muñoz et al., 2014). Many terrestrial and aquatic organisms have shifted their geographic distributions in response to changes in global temperature (Parmesan, 2006; Root et al., 2003). However, further study of the causal links between environmental temperature, physiology, and range size are necessary (Calosi et al., 2008; Chown, Gaston, & Robinson, 2004; Pörtner & Knust, 2007). For instance, if narrow‐ranging species are found to have less acclimation ability and/or lower tolerance to high temperature, increasing global temperatures could further contract and fragment their already restricted geographic distributions or increase pressure from competitors (Bernardo & Spotila, 2006; Gifford & Kozak, 2012; Jaeger, 1971). This could make them particularly vulnerable to extinction (Harris & Pimm, 2008; Mace et al., 2008). As ectotherms rapidly take on the temperature of their environment (Feder & Lynch, 1982; Hutchison, 1961; Lunghi et al., 2016), physiological plasticity may be especially crucial for survival.

It is anticipated that adaptation to local temperature gradients across the range should result in variation in acclimation ability within and between species (Angert, Sheth, & Paul, 2011; Davis, Shaw, & Etterson, 2005). Salamanders are found to conform quickly to the air temperature of their environment (Lunghi et al., 2016) and present an ideal model system to study associations between temperature, physiological tolerances, and range size. If individuals are rapidly adapting to thermal regimes, we should find a significant relationship between acclimation ability and temperature range of the environment at the population level of species (Bozinovic et al., 2011; Hereford, 2009; Snyder & Weathers, 1975).

Evolutionary trade‐offs in physiological tolerances may also play an important role in determining species’ range limits, yet are rarely investigated (Calosi et al., 2008; Pörtner et al., 2006; Stillman, 2003). For example, Stillman (2003) found that porcellanid crabs with the greatest thermal limits had the lowest acclimation ability for those traits, and suggested that trade‐offs should exist between acclimation capacity and thermal tolerance. Calosi et al. (2008), however, find that in European diving beetles, those species with the lowest acclimation ability also had the lowest tolerance to warm temperatures. Thus, it remains unclear whether general relationships between acclimation ability and thermal tolerance exist across taxa.

Here, we ask whether wide‐ranging species of North American salamanders have a greater propensity for thermal acclimation than narrow‐ranging species and whether a relationship between acclimation ability and environmental temperature range can be found within and among species. We focus on 16 ecologically similar salamander species from the family Plethodontidae. These species vary greatly in geographic range size and their evolutionary relationships are well resolved (Kozak, Mendyk, & Wiens, 2009), enabling results to be analyzed in a phylogenetic context. Using thermal tolerance data (measured as critical thermal maximum [CTMax]), we further test whether species with greater tolerance to heat have greater acclimation capacity at warmer temperatures, or whether as Stillman (2003) proposes, there are trade‐offs between these traits.

## 2 MATERIALS AND METHODS

### 2.1 Collection and maintenance of salamanders

Live salamanders were collected throughout the Appalachian Mountains of eastern North America from 2009 to 2012. Sixteen species of plethodontid salamanders representing four clades were examined: the genus *Desmognathus,* the *Plethodon cinereus* group, the *Plethodon glutinosus* group, and the *Plethodon wehrlei* group (Table 1). The family Plethodontidae represents a diverse group of lungless salamanders and the phylogenetic relationships among species in this study are strongly supported based on phylogenetic analysis of mitochondrial‐ and nuclear‐DNA sequences (Kozak et al., 2009). Species were chosen to represent a wide variety of range sizes, body sizes, and habitat types. *Plethodon* salamanders typically inhabit terrestrial habitats, whereas *Desmognathus* salamanders are more often found in association with seepages and streams (Petranka, 1998). Mature salamanders were collected from throughout the latitudinal extent of each species’ range.

**Table 1:** North American salamander groups and species, detailing number of individuals sampled (*n*), mean body mass, mean CTMax (critical thermal maximum), latitudinal extent, and mean acclimation ability

Species	*n*	Mean body mass (g)	Mean CTMax (°C)	Latitudinal extent (degrees)	Mean acclimation ability
*Desmognathus* group (6 species)					
* D. carolinensis*	8	1.10	32.2	1.26	−19.64
* D. fuscus*	20	2.07	33.1	13.65	20.66
* D. monticola*	24	3.66	33.1	9.12	35.66
* D. ochrophaeus*	36	1.01	33.1	8.94	9.68
* D. ocoee*	12	1.33	32.7	3.05	5.53
* D. orestes*	18	1.13	32.8	1.75	−23.35
*Plethodon cinereus* group (4 species)					
* P. cinereus*	44	0.89	32.5	14.28	6.56
* P. hubrichti*	11	1.18	32.1	0.07	−5.64
* P. richmondi*	14	1.07	32.7	3.59	−35.88
* P. virginia*	8	1.28	32.0	0.93	−24.92
*Plethodon glutinosus* group (4 species)					
* P. cylindraceus*	18	4.90	32.8	5.77	4.38
* P. glutinosus*	33	4.85	33.0	10.67	−19.60
* P. montanus*	22	1.95	31.7	1.84	−30.95
* P. teyahalee*	16	6.11	32.6	1.44	5.24
*Plethodon wehrlei* group (2 species)					
* P. punctatus*	6	3.81	31.9	1.66	−47.66
* P. wehrlei*	7	1.75	32.4	6.27	−10.97

Once collected, salamanders were maintained at 14°C in an environmental chamber until ready for testing. A light:dark photoperiod of 12L:12D was implemented. Salamanders were kept in individual plastic containers lined with moist paper towels and fed crickets on a weekly basis.

### 2.2 Experimental design (SMR measurements)

Conspecific salamanders were sorted by weight and then split randomly into two equivalent groups to be acclimated for a minimum of 3 weeks at 14°C or 22°C. Consistent with acclimation timelines in other amphibian studies, this time period should ensure full acclimation to these temperatures (see Feder, 1978; Feder, Gibbs, Griffith, & Tsuji, 1984; Hutchison, 1961). Environmental chambers maintained air temperature within 1°C of the desired acclimation temperature. Acclimation temperatures are representative of fairly typical late spring through early fall evening temperatures that surface‐active salamanders within each range would experience (Brattstrom, 1963; Feder & Lynch, 1982), although 22°C would be at the higher end for some montane endemics. Only sexually mature male and female salamanders were included in the analyses, however, gravid salamanders were not used. Juveniles were not considered as they have been found to have different physiological tolerances from adults in other amphibians (Lunghi, Manenti, & Ficetola, 2015; Spicer & Gaston, 1999). Sample sizes per species ranged from 6 to 44, with larger samples collected from across the range of wide‐ranging species and smaller samples from very restricted species. The summed total was 297 salamanders (see Table 1). Before each trial, salamanders were measured to the nearest 0.001 g.

To approximate SMR, we recorded oxygen consumption (VO_2_) at rest (see Fitzpatrick, Bristol, & Stokes, 1972; Homyack, Haas, & Hopkins, 2010). Automated closed‐system respirometry (Sable Systems International, Hendersonville, NV, USA) was used to measure oxygen consumption at three test temperatures of 5, 15, and 25°C. As diet influences metabolic rate, salamanders were not fed for 7 days prior to the start of the first respirometry trial (Feder et al., 1984; Lagerspetz, 1977). Oxygen consumption measurements were made at one temperature per day, with the order of test temperature assigned randomly to individuals and alternating every week. For each trial, a random assortment of salamander species were placed inside individual tubes (with two‐way stopcocks) within a digitally controlled incubator. Up to seven animals could be measured during the same trial, with each chamber recording independently. An empty chamber identical to the others was used as the baseline and control.

For each trial run, VO_2_ was recorded for individual chambers for 75 s at 10‐min intervals and then repeated for 2–3 hr. Air entering the salamander chambers was scrubbed of CO_2_ and entered at a flow rate of 250 ml/min. Before entering the respirometry tubes, air passed through a water air bubbler to control humidity and prevent desiccation of the salamanders. Air leaving the chambers passed through drierite and ascarite to remove both water vapor and CO_2_, before entering the oxygen analyzer where concentrations were recorded each second by data acquisition software (FC‐l0a, Sable Systems International, Las Vegas, NV, USA). Rates of oxygen consumption (μl O_2_/hr) were calculated based on equations from Withers (1977).

As SMR is meant to capture the metabolic rate of animals at rest (Fitzpatrick et al., 1972; Homyack et al., 2010), measurements were taken between 9 a.m. and 4 p.m., during the time when nocturnal salamanders would naturally be inactive. As salamanders inhabit underground retreats or spaces beneath rocks and logs during the day, they are well suited to dark, moist respirometer vessels. Test chambers were small enough to limit movement and salamanders are assumed to remain inactive for the most part (Feder et al., 1984). Salamanders were allowed to habituate inside the test chambers for the first hour, and as such, data from this period were not included in the analysis. From the remaining runs, the mean of the lowest two VO_2_ values was taken as the approximated SMR for each individual/test temperature.

Once all trials were complete for individuals at a given acclimation temperature, salamanders were given a minimum of 4 weeks at 14°C before being acclimated at the remaining temperature. Each individual, therefore, had a total of six trials (5, 15, and 25°C at each acclimation treatment of 14 and 22°C). Our experimental design attempted to control for many of the extrinsic factors that can affect metabolic rate, for example, feeding, photoperiod, acclimation length, activity, season, and reproductive state (see Feder et al., 1984; Homyack et al., 2010; Lagerspetz, 1977). Using data from individuals, average SMRs were calculated for each species.

### 2.3 Weight correction

As metabolic rate is influenced by body mass, we corrected VO_2_ data for differences in weight prior to running all analyses to ensure a more accurate comparison of individuals of different masses. We used the formula *M* = *A *× *W* ^*b*^, where *M* = metabolic rate in μl O_2_ consumed per hour; *W* = body weight in g; and *A* and *b* are constants. The constant *A* is the “intercept” of the line and relates to the amount of oxygen consumed by an organism of unit weight, whereas constant *b* reflects the degree to which metabolic rate is affected by body mass, and is the slope of the line for the log_10_‐log_10_ plot of SMR versus mass (Feder, 1976). Slopes for this relationship depend on test temperature. Calculated average slopes used in the equation are *b* = 0.59 for 5°C and *b* = 0.71 for measurements at 15 and 25°C.

### 2.4 MCMCglmm statistical analyses

To determine whether there are differences in SMR between wide and narrow‐ranging species when acclimated at different temperatures, we ran a phylogenetically controlled Markov chain Monte Carlo generalized linear mixed model (MCMCglmm) with repeated measures (Hadfield, 2010). MCMCglmm uses a Bayesian approach to fit general linear models and includes the phylogenetic variance–covariance matrix as a random effect in the regression model, allowing for any genetic influences in the data to be accounted for.

All modeling was conducted in R ver. 3.1.2 (R Core Team, 2013) using packages “ape” (Paradis, Claude, & Strimmer, 2004) and “MCMCglmm” (Hadfield, 2010); see Appendix S1 for R code. Fixed factors to examine the influence on VO_2_ included: test temperature (5, 15, 25°C), acclimation temperature (14 and 22°C), and range size (latitudinal extent, as well as species grouped as wide vs. narrow‐ranging). To assign species as wide or narrow‐ranging, a natural break was found between species with latitudinal extents greater than or less than five degrees of latitude. Sex and genus were also included as covariates in the model. As *Desmognathus* salamanders are often associated with streams and seeps, they could be better buffered from temperature extremes than more terrestrial *Plethodon* salamanders. This could have a potential influence on salamander physiological tolerances and SMR. The covariate “genus” therefore divides *Plethodon* from *Desmognathus* to help to control for differences in thermal habitat associated with each group.

As individual salamanders were used in multiple trials, an additional random effect was included in the model to account for repeated measures of individuals. The initial model included several interactions (test temperature × acclimation temperature, test temperature × range size, acclimation temperature × range size, and test temperature × acclimation temperature × range size), however, the three‐way interaction, as well as the two‐way interactions for test temperature × acclimation temperature and test temperature × range size and had *p*‐values >.3 and were removed from the model. Our priors took the form of: prior <‐list(G = list(G1 = list(V = diag(2), nu = 2, alpha.mu = c(0,0), alpha.V = diag(2) × 1,000)), R = list(V = diag(1), nu = 0.002)), where a 2 × 2 covariance matrix is being estimated for the random effects (G) and a scalar variance for the residuals (R). Using trace plots, we observed the distribution of samples to remain stationary over time, therefore giving us confidence that our posterior is a good approximation of the true distribution. For optimal outcomes, we ran the analysis for 300,000 iterations, with 25,000 samples of burn‐in, and sampling every 1,000th generation.

Further, additional MCMCglmm analyses were performed where data were grouped separately by range size into wide and narrow‐ranging species. This grouping enabled examination of the affect of acclimation temperature on SMR within each group (wide and narrow‐ranging) and for each test temperature (5, 15, 25°C). Acclimation (14 and 22°C) was included as a fixed factor, while sex remained as a covariate. *T*‐tests were also performed on each species for each test and acclimation temperature, to determine which species showed evidence of thermal acclimation or metabolic depression.

### 2.5 Phylogenetic consideration of remaining tests

Remaining statistical analyses do not have the phylogeny incorporated into the model as in earlier MCMCglmm analyses. As phylogenetic non‐independence may influence results in comparative analyses of multiple species, we need to test for the phylogenetic influence of measured traits (e.g., acclimation ability, CTMax, thermal range, latitudinal extent). Lambda tests were employed using “Fit Continuous” model tests in the “geiger” package of R (v.3.0.2, R Development Core Team, 2013). In all cases, lambda was chosen as the best model with lambda scores for traits between 0.00 and 0.086. These low lambda values indicate that these particular traits have very little phylogenetic signal, enabling us to use the original data without further concern for phylogenetic influence.

### 2.6 Acclimation ability and thermal range

To better assess the relationship between physiological tolerances and local thermal environments, we estimated the thermal range of localities for each species and performed linear regressions between acclimation ability versus environmental thermal range and acclimation ability versus latitudinal extent. Acclimation ability was defined as any positive increase in VO_2_ from lower to higher acclimation temperatures, using VO_2_ at 22°C acclimation minus VO_2_ at 14°C acclimation (Table 1). Any increase in VO_2_ for individuals acclimated at a higher temperature indicates acclimation capacity, whereas any decrease was considered metabolic depression. Only SMR data at the 25°C test temperature were used to determine warm temperature acclimation ability, as this is where we find the greatest influence of temperature on acclimation.

To approximate the annual thermal range of each locality sampled, thermal data (averages 1950–2000) were obtained from the Worldclim online database at 1 km^2^ resolution (Hijmans, Cameron, Parra, Jones, & Jarvis, 2005). The program DIVA‐GIS (Hijmans, Guarino, & Rojas, 2002) was used to georeference and map salamander localities. Data were then extracted for the bioclimatic variable Bio 7 (temperature annual range), which represents air temperature of the local area. Salamanders are found to conform quickly to the temperature of their environment and air temperature has been found to be a good proxy of operative conditions actually experienced by terrestrial salamanders (Lunghi et al., 2016). Genus (*Plethodon* vs. *Desmognathus*) was included as a covariate.

Finally, intraspecific regressions were performed to test how acclimation ability is influenced by natural thermal regimes experienced by populations. Nine of 16 species had data for multiple localities across the geographic range and could be used to test for relationships between temperature range of the environment and acclimation ability.

### 2.7 Physiological trade‐offs

As species that have evolved the greatest tolerances to high temperatures may have done so at the expense of acclimation capacity (Stillman, 2003), we further tested whether there is a trade‐off between CTMax and thermal acclimation ability in these species of salamanders. Critical thermal maximum data were obtained using methods similar to Layne and Claussen (1982), where loss of righting response is considered the endpoint. This point is reached when a salamander is turned onto its back and is unable to right itself within 30 s (Hutchison, 1961). At the start of each trial, individual salamanders were placed into a small container with 2 cm of water and an open top. A 150‐watt infrared‐heat lamp was placed 27 cm from the surface of the water and increased water temperature by 0.5°C/min until the end point was reached. An air bubbler was used to circulate the water. Deep body temperatures of salamanders are found to closely follow water temperature at rates of 1.0°C/min or less (Feder & Lynch, 1982; Hutchison, 1961), therefore, the water temperature at the endpoint of each trial was considered the CTMax result for that individual (as measured by digital thermometer; Fluke 51 II, Everett, WA, USA) (Brattstrom, 1968; Lutterschmidt & Hutchison, 1997). Further, to reduce the affect of diet on thermal physiology, salamanders were not fed for 6 days prior to measurements of CTMax (Hutchison, 1961). Each salamander was weighed before the trial to within 0.001 g.

Using linear regression, we then examined whether there was any indication of a trade‐off between CTMax and warm temperature acclimation ability. As warm test temperatures are most relevant to examining warm temperature acclimation response, we focused on individual‐level acclimation differences in VO_2_ at the 25°C test temperature. Sex was included as a covariate.

## 3 RESULTS

**Figure 1:**
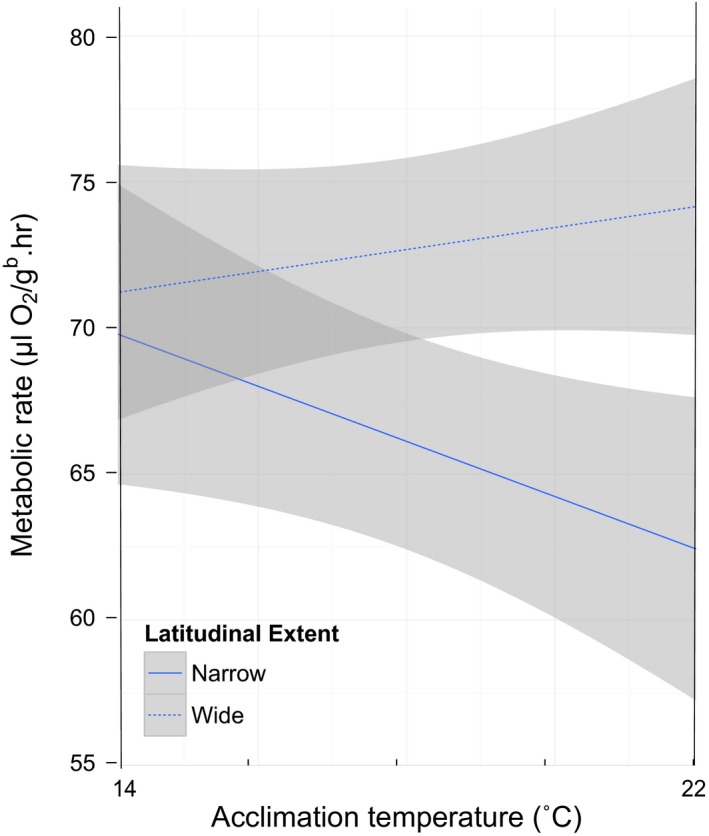
Acclimation temperature × latitudinal extent, two‐way interaction from MCMCglmm model (*p *<* *.004, *n* = 16 species). Wide‐ranging North American salamander species increase metabolic rate at a higher acclimation temperature, whereas narrow‐ranging species have a sharp drop in metabolic rate. Metabolic rates are weight‐corrected. Shaded areas indicate 95% confidence intervals

Our MCMCglmm analyses of the full data set revealed significant two‐way interactions between acclimation temperature and geographic range size (*p *<* *.004 for both latitudinal extent and wide vs. narrow‐ranging species). Results show that wide‐ranging salamander species increase metabolic rates from low to high acclimation temperatures, whereas narrow‐ranging species have a drop in SMR at the higher acclimation temperature (Figure 1). Sex remained as a significant covariate in both models (*p *<* *.025), as did genus (*p *<* *.004), yet neither had a major influence on the overall results.

**Figure 2:**
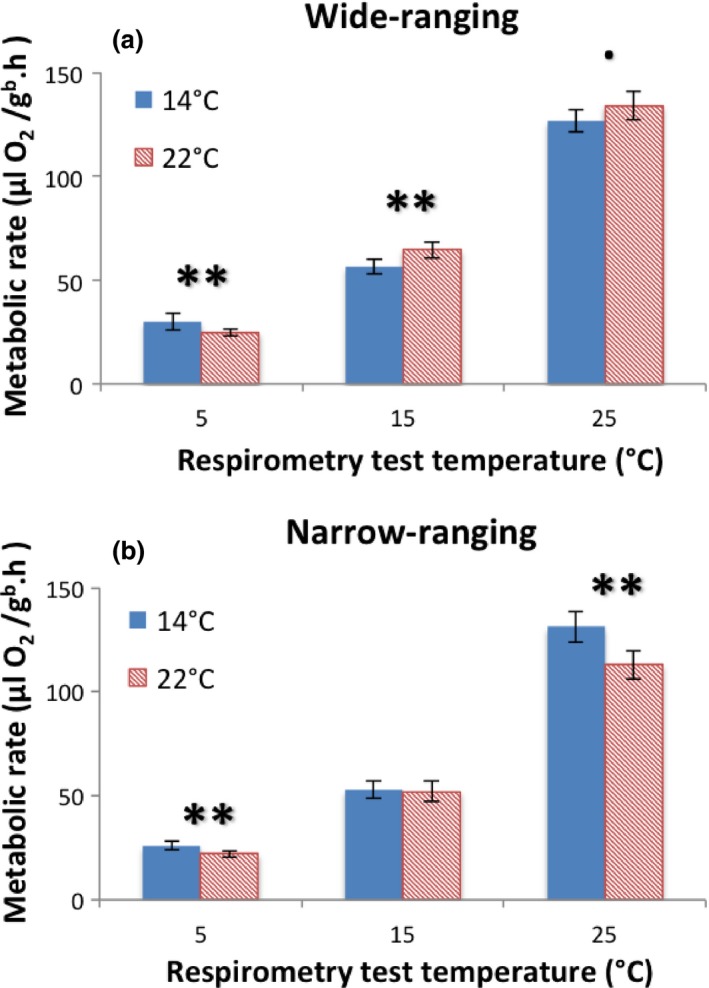
Affect of acclimation temperature on weight‐corrected standard metabolic rates of North American salamanders. Species grouped into (a) wide and (b) narrow‐ranging based on latitudinal extent > or <5° (16 species: seven wide and nine narrow‐ranging). MCMCglmm to test for differences in VO_2_ between acclimation temperatures for each test temperature. Means are plotted ± 95% confidence limits. Significance of difference between 14 and 22°C is given above each test temperature: ● = *p *≤* *.1, * = *p *<* *.05, ** = *p *<* *.01; no symbol = *p *>* *.1

When tested within range size group, MCMCglmm analyses reveal that for wide‐ranging species acclimated at the higher temperature, there is a significant decrease in metabolic rate at the 5°C test temperature (*p *<* *.004) and a significant increase in metabolic rate at the 15°C test temperature (*p *=* *.0073) (Figure 2a) compared to those acclimated at a lower temperature. At the 25°C test temperature, there is no change in SMR for salamanders acclimated at the higher temperature, with the *p*‐value falling just outside of the significance level (*p *=* *.058). While the overall trend for wide‐ranging species is a slight increase in metabolic rate for species acclimated at the higher temperature of 22°C, the differences were minor and not significant at the 25°C test temperature. Narrow‐ranging species acclimated at 22°C are found to have significantly lower metabolic rates at test temperatures 5°C (*p *<* *.004) and 25°C (*p *<* *.004) compared to those acclimated at 14°C. The overall trend is for decreased metabolic rate for narrow‐ranging salamanders acclimated at the higher temperature, and this is quite pronounced at the highest test temperature (Figure 2b).

At the species‐level, SMR results and *t*‐tests revealed some trends of interest (Table 2), with results of particular interest being found at the higher test temperatures (15 and 25°C), as this is where there is the greatest response of warm temperature acclimation. For wide‐ranging species, the majority of species are found to show little difference in metabolic rate between acclimation temperatures. *D. monticola* acclimated at 22°C, however, has a statistically significant increase in SMR at the 25°C test temperature (*p *=* *.00039). For the narrow‐ranging species, four of the nine species (*D. orestes, P. montanus, P. punctatus,* and *P. richmondi*) acclimated at 22°C show a significant drop in SMR at the 25°C test temperature (*p*‐values all < .05). These results indicate that several of the narrow‐ranging species in this study appear to be very sensitive to warm temperatures and exhibit metabolic depression, whereas no wide‐ranging species have a significant drop in metabolic rate at higher test/acclimation temperatures.

**Table 2:** Weight‐corrected standard metabolic rates (μl O_2_/hr) of North American salamander species at each test temperature (TT) and acclimation temperature (AT) ± standard error

Species	TT = 5C		TT = 15C		TT = 25C	
	AT = 14C	AT = 22C	AT = 14C	AT = 22C	AT = 14C	AT = 22C
*D. carolinensis*	28.27 ± 11.29	19.17 ± 7.36	60.27 ± 19.91	85.43 ± 26.53	129.4 ± 28.26	117.53 ± 24.71
*D. fuscus*	35.02 ± 6.89	34.7 ± 6.92	77.71 ± 16.11	90.33 ± 14.78	128.1 ± 18.28	145.0 ± 26.69
*D. monticola*	30.37 ± 5.63	18.14 ± 4.26	65.09 ± 6.31	73.91 ± 7.7	111.0 ± 11.09	140.8 ± 13.03
*D. ochrophaeus*	26.02 ± 3.61	23.99 ± 3.49	64.94 ± 7.65	77.41 ± 9.17	134.4 ± 7.91	140.7 ± 14.63
*D. ocoee*	29.17 ± 7.65	32.30 ± 4.88	59.81 ± 13.85	71.82 ± 18.02	144.8 ± 30.52	154.3 ± 30.59
*D. orestes*	27.16 ± 5.88	29.46 ± 3.31	73.05 ± 11.84	60.29 ± 11.39	129.6 ± 19.02	98.96 ± 17.13
*P. cinereus*	43.13 ± 15.9	24.41 ± 2.91	53.47 ± 7.98	60.1 ± 8.59	137.1 ± 13.9	152.9 ± 16.98
*P. cylindraceus*	22.15 ± 4.24	29.8 ± 6.45	44.25 ± 6.72	50.30 ± 7.54	110.5 ± 8.75	114.9 ± 12.26
*P. glutinosus*	22.73 ± 3.55	21.73 ± 3.98	47.19 ± 4.86	49.09 ± 6.2	123.0 ± 15.32	105.1 ± 10.04
*P. hubrichti*	34.39 ± 9.87	26.88 ± 5.58	38.83 ± 6.19	52.07 ± 20.88	148.9 ± 32.2	133.2 ± 26.37
*P. montanus*	22.43 ± 4.04	21.14 ± 3.22	53.14 ± 8.35	45.17 ± 7.04	125.8 ± 20.84	93.88 ± 18.12
*P. punctatus*	19.46 ± 3.31	26.13 ± 6.41	37.33 ± 14.53	43.69 ± 10.89	149.9 ± 30.28	111.4 ± 16.21
*P. teyahalee*	24.92 ± 6.24	25.29 ± 5.04	48.1 ± 10.19	48.68 ± 10.6	118.2 ± 18.63	119.9 ± 11.8
*P. richmondi*	21.48 ± 3.43	18.46 ± 7.45	39.71 ± 5.06	24.57 ± 4.55	110.6 ± 12.83	89.19 ± 13.91
*P. virginia*	31.2 ± 10.07	20.8 ± 6.35	52.24 ± 21.65	46.69 ± 15.51	160.6 ± 43.05	136.7 ± 43.37
*P. wehrlei*	13.8 ± 4.99	27.58 ± 2.58	30.39 ± 9.39	38.24 ± 6.88	132.7 ± 31.07	123.5 ± 48.23

Weight correction follows formula *M* = *A *× *W* ^*b*^, where *M* = metabolic rate in μl O_2_ consumed per hour, *W* = body weight in g, and *A* and *b* are constants.

**Figure 3:**
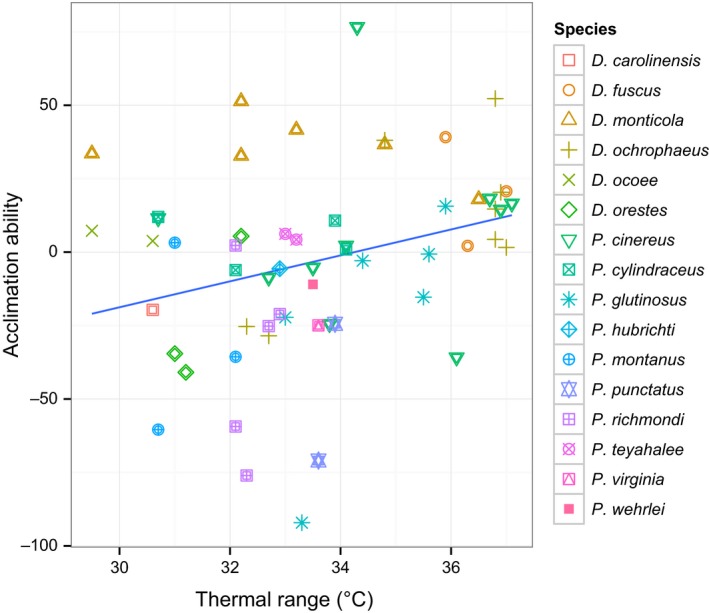
Relationship between acclimation ability and thermal range of the environment (°C) for 16 species of Plethodontid salamanders at the population level (*p = *.027, *R* ^2^ = .18). Acclimation ability calculated as VO_2_ at 22°C acclimation—VO_2_ at 14°C acclimation for the 25°C test temperature. Standard metabolic rates were weight‐corrected prior to the analysis. Thermal range data (temperature annual range) were obtained from the Worldclim online database

**Figure 4:**
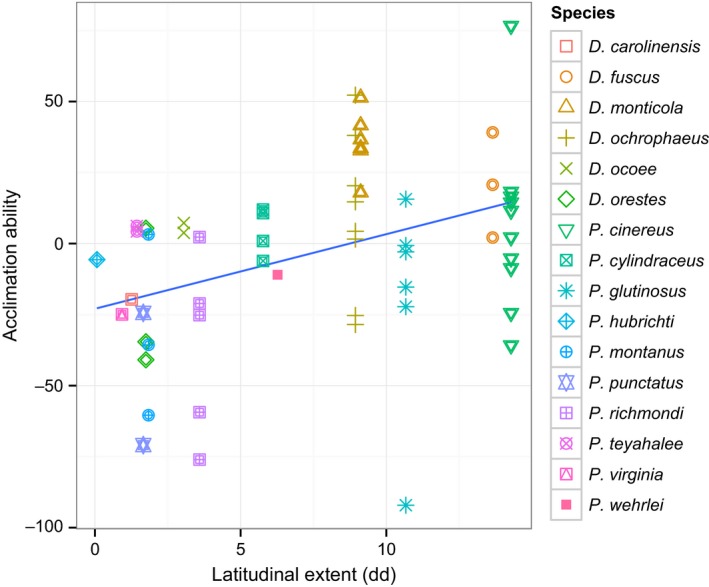
Relationship between mean acclimation ability and latitudinal extent (decimal degrees) for 16 species of salamanders at the population level (*p = *.0018, *R* ^2^ = .25). Acclimation ability calculated as VO_2_ at 22°C acclimation—VO_2_ at 14°C acclimation for the 25°C test temperature. Standard metabolic rates were weight‐corrected prior to the analysis

For acclimation ability versus thermal range of the environment across all species at the population level, we find there to be a significant positive relationship (*p = *.027, *R* ^2^ = .18), where mean acclimation ability of populations increases with increasing temperature range of the environment (Figure 3). In addition, mean acclimation ability of species is correlated with latitudinal extent (*p = *.0018, *R* ^2^ = .25, Figure 4). Genus (*Plethodon* vs. *Desmognathus*) was included as a covariate in both analyses and was found to be significant (*p = *.0044 and *p = *.0025, respectively). For *Plethodon* species, there is found to be a stronger association between acclimation ability and temperature range than in *Desmognathus* species.

For population‐level tests within salamander species, only one of nine species tested (*D. ochrophaeus*), is found to have a significant positive relationship between acclimation ability and temperature range of the local environment (*p = *.035, *R* ^2^ = .55). No intraspecific relationships are found for the other eight species.

Finally, we find a positive relationship between acclimation ability and CTMax (*p *=* *.009), where species with the poorest acclimation response to warm temperature also have the lowest tolerances to heat. Sex was found to be a significant covariate (*p *=* *.029) and remained in the model. Critical thermal maximum ranged from 31.7°C in *P. montanus* to 33.1°C in *D. fuscus, D. monticola,* and *D. ochrophaeus* (Table 1).

## 4 DISCUSSION

Acclimation ability and thermal tolerance are key traits in physiological ecology and could play an important role in shaping the geographic ranges of species (Bozinovic et al., 2011; Calosi et al., 2008; Spicer & Gaston, 1999; Sunday et al., 2011). If acclimation ability is correlated with environmental thermal variability, then species experiencing broader thermal variation (i.e., wide‐ranging species) should have a greater capacity for thermal acclimation (Brattstrom, 1968; Feder, 1978). Results of our MCMCglmm analyses indicate such an association, where wide‐ranging species of temperate salamanders are found to have a greater propensity for thermal acclimation than narrow‐ranging species.

Associations between environmental temperature range and physiological tolerances have been found in many species (Addo‐Bediako et al., 2000; Angilletta, 2009; Calosi, Bilton, Spicer, Votier, & Atfield, 2010; Sunday et al., 2011), with taxa including fish, insects, birds, and amphibians having demonstrated correlations between habitat thermal variability and capacity for thermal acclimation (Brattstrom, 1968; Hoffmann & Watson, 1993; McKechnie, 2008; Prosser, 1975). For thermal acclimation of metabolism in particular, several species of wide‐ranging temperate amphibians have shown some degree of acclimation (Feder, 1978; Fitzpatrick, 1973; Fitzpatrick & Atebara, 1974; Fitzpatrick, Bristol, & Stokes, 1971; Fitzpatrick et al., 1972), whereas two neotropical salamander species were found to have metabolic depression at a higher acclimation temperature (Feder, 1978). Similar to our results, these studies suggest that thermal acclimation is most prevalent among species experiencing higher degrees of environmental temperature variation. While the acclimation tendency of many temperate zone salamanders is to retain a relatively homeostatic metabolic rate at different temperatures, increased SMR may be also advantageous at times where energy expenditure can lead to acquisition of greater resources. (Bernardo et al., 2007; Feder, 1976, 1978; Hillman et al., 2009). For instance, higher SMR can encourage greater vagility and genetic exchange (Bernardo et al., 2007). Acclimation ability could, therefore, have been a distinct advantage for some species in facilitating range expansion (Angilletta, 2009; Hillman et al., 2009), thus supporting predictions of the climate variability hypothesis (Calosi et al., 2008; Stevens, 1989). Even among temperate species, our study demonstrates that there is seemingly enough seasonal variation throughout the landscape to select for species with greater acclimation.

Conversely, many temperate amphibians (including salamanders) are adapted for activity at mild/cooler temperatures and have field body temperatures below 20°C (Brattstrom, 1963; Feder & Lynch, 1982; Kozak & Wiens, 2007). Physiological specialization to cooler habitats has been hypothesized to constrain the evolution of broad geographic ranges and to limit lowland dispersal (Bernardo & Spotila, 2006; Gilchrist, 1995; Huey & Kingsolver, 1993). Although some species of *Plethodon* inhabit a wide range of elevations, recent work suggests that the ancestor of this group was restricted to a montane climate (Kozak & Wiens, 2010). Thermal acclimation would have little value in more stable thermal environments, and selection for acclimation ability would be discouraged if it comes at a cost (Feder, 1978). Therefore, the relative stability of higher elevations may limit opportunities for cool‐adapted salamanders to expand into warmer habitats (Gifford & Kozak, 2012).

Narrow‐ranging montane species, in particular, are seemingly more sensitive to warm temperatures, and several species in this study were found to suffer metabolic depression at the higher test and acclimation temperatures. In fact, there are already indications that some montane salamander species are living in temperatures that reach the limits of their physiological tolerances (Bernardo & Spotila, 2006; Gifford & Kozak, 2012). For species with limited tolerance to warm temperatures, even moderate increases in heat can have large affects on physiological systems. High temperatures can impair enzymatic function and disrupt membrane structure, causing decreased locomotion ability, digestive inefficiencies, and reduced growth (Angilletta, 2009; Hillman et al., 2009). For lungless salamanders, cutaneous respiration on its own may not provide sufficient oxygen at warmer temperatures (Whitford, 1973; Whitford & Hutchison, 1965). Ultimately, warming‐induced stress can lower the fitness and survival of individuals. The inability of species to disperse across warm, dry valleys may further increase the risk of extinction for narrow‐ranging montane species. Not only is the suitable habitat of their current geographic ranges likely to contract with global temperature rise, but impeded dispersal can lead to genetic erosion and population decline (Bernardo & Spotila, 2006; Calosi et al., 2008; Gifford & Kozak, 2012; Parmesan, 2006). Reductions in surface activity may offer some reprieve from temperature extremes, but salamanders may be especially sensitive to rapid or prolonged changes in their environment (Huey & Stevenson, 1979). Further, with climate change the geographic ranges of competitors and predators may extend further upslope into the ranges of montane species, adding pressure to species already under stress (Bernardo & Spotila, 2006; Gifford & Kozak, 2012). How a species will ultimately fare when faced with global temperature rise will depend on the plasticity and strength of their thermal tolerances, their ability to adapt, and their accessibility to alternative suitable habitat (Davis & Shaw, 2001; Kozak & Wiens, 2010).

Physiological tolerances are expected to evolve quite rapidly under selection to environmental temperature, and should be differentiated across a latitudinal range (see Bozinovic et al., 2011; Hereford, 2009; Hurme, Repo, Savolainen, & Paakkonen, 1997; Rehfeldt et al., 2002; Snyder & Weathers, 1975). While we find evidence for this when data are combined for all species, within species, there are few clear relationships between acclimation ability and thermal regimes. The only species to demonstrate a significant relationship between acclimation capacity and thermal range is *D. ochrophaeus*. While we did not sample the entire latitudinal extent of these species, we still fail to see a clear pattern in wide‐ranging species such as *P. cinereus* where 10 populations were tested and thermal range varies by over 7°C between populations. As salamanders have very small home ranges and low dispersal rates (Petranka, 1998; Wells, 2007), there is good potential for rapid local adaptation. However, studies of amphibians and other taxa (Brattstrom, 1968; Hoffmann & Watson, 1993) have also found little within species variation in thermal tolerances. The lack of among‐population variation could potentially be the result of the dynamic climatic history in North America, offering opportunities for both range expansions and contractions (Parmesan et al., 2005; Pielou, 1991). The New World temperate zone is the center of origin for plethodontids, with the clade more recently colonizing and radiating in the New World tropics (Wake & Lynch, 1976; reviewed in Kozak, 2017). Plethodontid lineages that radiated rapidly into different thermal regimes could have done so by gaining adaptive physiological ability over other close relatives with similar ecologies. Therefore, rapid expansion of leading‐edge populations with broad tolerances may help to explain the lack of among‐population variation within wide‐ranging species (Sage & Wolff, 1986; Zink & Dittmann, 1993).

Although temperature is thought to be a critical factor in limiting species’ distributions in North America (Merriam, 1984; Root, 1988; Whitton, Purvis, Orme, & Olalla‐Tárraga, 2012), our analysis did not consider other variables known to influence geographic ranges in this group. For instance, other habitat requirements (e.g., precipitation), and biotic factors including competition could also limit species’ ranges and thereby contribute to variation in range size. However, for salamanders, precipitation alone was not found to be a significant predictor of distributions in other studies looking at salamanders (see Quintero & Wiens, 2013 *)*, and interspecific interactions have only been found to limit portions of distributions (Deitloff, Church, Adams, & Jaeger, 2009; Gifford & Kozak, 2012; Jaeger, 1971). As many of the species in this study are found to coexist regionally and locally (Adams, 2007; Highton, 2004), it suggests that biotic interactions do not underlie variation in geographic range size in the clades we examined. While temperature and physiology alone are not likely to explain all of the aspects of a species’ range, there is empirical support for the strong influence of these factors in determining geographic distributions (Addo‐Bediako et al., 2000; Calosi et al., 2010; Gaston, 2003; Hillman et al., 2009). In addition, we also did not investigate seasonal and developmental plasticity of these traits, which could potentially influence the extent of the response (see Hoffmann, Tomiuk, Schmuths, Koch, & Bachmann, 2005; Terblanche & Chown, 2006).

Another consideration includes differences in habitat use between species that may contribute to disparities in temperature experienced. Most *Desmognathus* species are associated with riparian habitats and may be buffered from thermal extremes. To control for this, genus (*Plethodon* vs. *Desmognathus*) was added as a covariate to several of the analyses. While genus is found to be a significant factor, the overall results remain unchanged when it is included. Between the two groups, there is found to be a stronger association between acclimation ability and temperature range for *Plethodon* over *Desmognathus* species. However, the data points for *Desmognathus* salamanders are not found to be outliers and follow the expected pattern of more terrestrial salamanders. The lesser association does indicate though that some temperature buffering may be occurring in certain individuals/populations of *Desmognathus* salamanders.

Finally, one question that remains is whether there are any evident trade‐offs or relationships in physiological traits for these species of salamanders. Stillman (2003) proposes that species that have evolved the greatest tolerances to high temperatures may have done so at the expense of acclimation capacity. However, similar to the results of Calosi et al. (2008), we find that species with the lowest tolerances to warm temperatures (measured as CTMax) also have the poorest acclimation ability to warm temperatures. This relationship suggests that both traits are potentially good predictors of how a salamander species may respond to climatic change and further highlights the vulnerability of narrow‐ranging salamanders to global temperature increase (Calosi et al., 2008). Not only do these species have lower tolerances to high temperatures, but they also have a lesser ability to acclimate to a warming climate.

## 5 CONCLUSIONS

In this study, we examined the relationship between physiological variation and the disparity in geographic range sizes exhibited among closely‐related species of salamanders. Thermal acclimation may enable a species to occupy more seasonal habitats, and is thought to be an important factor determining life histories and distributions of species (Angilletta, 2009; Feder, 1978). Some even argue that acclimation ability may be more important than thermal tolerances in determining vulnerability to climate change (Stillman, 2003). Here, we find a significant difference in acclimation ability between wide and narrow‐ranging temperate salamanders. Wide‐ranging species maintain or increase metabolic rate when acclimated at a higher temperature, whereas many narrow‐ranging species show significant metabolic depression at higher test and acclimation temperatures. In addition, we find a significant relationship between acclimation ability and environmental thermal range at the species‐level, but little evidence at the population level within species. These strong species‐level results support predictions of the climate variability hypothesis and indicate that acclimation ability could have played a major role in the latitudinal range expansion of wide‐ranging salamander species. Finally, as narrow‐ranging salamander species are found to have lower tolerance to high temperature, low acclimation ability, restricted distributions, and little access to alternative habitats, they are likely at higher risk of extinction in association with anthropogenic climate warming.

## CONFLICT OF INTEREST

None declared.

## AUTHOR CONTRIBUTIONS

T.M. and K.H.K. conceived the ideas; T.M. and K.H.K collected the specimens; T.M. collected and analyzed the physiological data; T.M. contributed the majority of the writing. Both authors contributed critically to the drafts and gave final approval for publication.
