# Supplementary material for: Low acclimation capacity of narrow‐ranging thermal specialists exposes susceptibility to global climate change
Source: Ecol Evol. 2018 Apr 15;8(9):4644–56. doi: 10.1002/ece3.4006 (PMC5938462; doi:10.1002/ece3.4006)
Supplement: Supplementary file 1 [file ECE3-8-4644-s001.docx]

**Supporting Information**

**Appendix S1: MCMCglmm R Code**

install.packages("ape")

install.packages("MCMCglmm")

library(ape)

library(MCMCglmm)

dataset<-read.csv(file="RespData.csv", head=TRUE)

attach(dataset)

dataset$Range<-as.factor(dataset$Range)

str(dataset)

#Phylogeny Component

tree<-read.tree("Salamander_phylogeny.phy")

species<-c("D._carolinensis", "D._fuscus", "D._ochrophaeus", "D._ocoee", "D._orestes", "D._monticola", "P_cinereus", "P_cylindraceus", "P_glutinosus", "P_hubrichti", "P_montanus", "P_punctatus", "P_richmondi", "P_teyahalee", "P_virginia", "P_wehrlei")

pruned.tree<-drop.tip(tree,tree$tip.label[-match(species, tree$tip.label)])# Tree pruned to include only species of interest from larger data set

sptree<-makeNodeLabel(pruned.tree, method="number", prefix="node") #rename nodes to be unique

treeAinv<-inverseA(sptree, nodes="TIPS")$Ainv

prior<-list(G=list(G1=list(V=diag(2), nu=2, alpha.mu=c(0,0), alpha.V=diag(2)*1000)), R=list(V=diag(1), nu=0.002))

random=~us(1+Temp):ID

#Final Model: Range size (Range)

model<-MCMCglmm(VO2_wc~1+Range+Acclm+Temp+Sex+Acclm*Range, random=random, data=dataset, family="gaussian", ginverse=list(species=treeAinv), prior=prior, nitt=300000, burnin=25000, thin = 1000, verbose=FALSE)
